# Development of Monoclonal Antibodies for Identifying Plant-Parasitic Nematodes

**DOI:** 10.2478/jofnem-2025-0024

**Published:** 2025-07-19

**Authors:** M. Bogale, E. Sampson, W. Hu, A. Baniya, S. Mishra, K. Kwon, H. D. Lopez-Nicora, P. DiGennaro

**Affiliations:** University of Florida, Gainesville, FL 32611; The Ohio State University, Columbus, OH 43210; University of California-Riverside, Riverside, CA 92521; University of Wisconsin-Madison, Madison, WI 53706

**Keywords:** HG type, monoclonal antibody, race, single-cell sequencing

## Abstract

Currently available nematode identification techniques rely on visual microscopic examination of their morphology and limited molecular assays. These methods generally serve their purpose of enumerating nematode genera and informing management recommendations. However, when identifying variations in pathogenicity or virulence within nematode populations and species – which is crucial for specific plant-parasitic nematode management recommendations – these methods are insufficient. Here, we demonstrate that nucleotide sequence information for tens of thousands of monoclonal antibodies (mAbs) can be generated for identification purposes using a single-cell RNA-seq of mature B cells obtained from mice immunized with nematode antigens. We also provide proof of concept by synthesizing two of these mAbs in vitro and demonstrate specificity using ELISA. Since mAbs can bind to a variety of molecules, their potential use may surpass discrimination among pathotype groups and shed light on what contributes to pathogenicity or virulence of nematodes that produce, or are associated with, these antigenic molecules.

Nematodes are among the most diverse metazoans, comprising nearly 30,000 described and an estimated 0.5–10 million undescribed (Hodda, 2022) species. Though plant-parasitic nematodes (PPN) that have been described thus far (~4,100 species; Decraemer and Hunt, 2006) constitute a small fraction of these species, PPN cause a significant amount of economic loss (over USD 80 billion/annum) to the global economy (Jones et al., 2013; Nicol et al., 2011). In soybean, the most economically damaging pests are the soybean cyst nematode (SCN; *Heterodera glycines*) and root-knot nematode (RKN; *Meloidogyne* spp.), which significantly reduce crop yield (Westphal and Xing, 2006). Thus, accurate identification of these nematodes is of great significance for improved management of these pathogens. Currently, soil samples are tested for the presence of PPN, including SCN and RKN, via soil extraction and enumeration based on nematode morphology, coupled with limited molecular diagnostic assays; however, these methods can only test for the presence or absence of PPN, generally at the genus level, with some relative quantification. This information, while valuable, may be insufficient for growers looking to make informed decisions on crop or cultivar choice. Different populations of SCN (HG types) or RKN (races) can display significantly different pathogenicity (Sturhan, 1972; Niblack et al., 2002). The designations of HG type (for SCN) and race (for RKN) describe pathogenicity at the population level. Importantly, these descriptors are of nematode populations, such that no two nematode populations can be described as genetically, or antigenically, identical; this is because some nematode populations may exist as a single pure pathotype, while others may contain several pathotypes or virulence phenotypes at different frequencies (Montarry et al., 2020).

Other shortcomings of classic morphology-based nematode identification include the availability of only a limited number of morphological characteristics to distinguish a vast number of species; the need for highly-trained personnel that are in short supply; the presence of cryptic species; and need for pathotype identifications. Various molecular diagnostic tools have been developed to supplement and/or circumvent these problems (Bogale et al., 2020). Each of these tools has, however, its own advantages and limitations. One of the earliest supplementary methods developed for the identification of PPN used serological techniques (Bird, 1964; Schots et al., 1989). Serological tools — more specifically, monoclonal antibodies (mAbs) — were also employed in the detection, identification and quantification of other plant pathogens, including viruses, bacteria, and fungi (Thornton, 2007; Arie et al., 1991; Arie et al., 1995).

The development of these mAb tools was largely dependent on the hybridoma technique (Köhler and Milstein, 1975), in which nematode-specific antibody-producing B cells are fused with cancer cells to immortalize them for a constant supply of the antibodies in culture. The hybridoma technique has, however, proved to be cumbersome and inefficient due to the low proportion of successful fusions obtained between B and tumor cells. Consequently, the use of mAbs for identification purposes has not been pursued much further, despite its potential.

In this study, we describe single-cell B cell receptor sequencing (scBCR-seq) of mature B cells obtained from mice immunized with nematode-derived antigens — allowing for the generation of tens of thousands of potentially unique mAb-encoding nucleotide sequences. We also provide proof of concept by synthesizing two mAbs using a cell-free protein synthesis system (CFPS), and testing these antibodies for specificity using enzyme-linked immunosorbent assay (ELISA). Since mAbs can bind with high specificity to a wide range of protein and non-protein targets such as nucleic acids, lipids, polysaccharides, and secondary metabolites (Haji-Ghassemi et al., 2015; Alving, 2006; Visan, 2018), these mAbs may shed light on what contributes to the differences in virulence that has been observed among different populations within a PPN species.

## Materials and Methods

### Immunization of mice with nematode antigen pools

Four nematode antigens were prepared by grinding masses of eggs (for root-knot nematode [RKN; *Meloidogyne incognita*] races 3 and 4) and egg bearing cysts (for soybean cyst nematode [SCN; *Heterodera glycines*] HG types 0 and 1.2.3.5.6.7) in liquid nitrogen using mortar and pestle. Since grinding eggs and cysts into powder was impossible, suspensions were made from pulverized nematode material, which were then withdrawn using 29-gauge, U-100 insulin syringe (Smiths Medical International Ltd., Keene NH, USA) to immunize mice. Immunization, cytometry and single-cell RNA-seq (scRNA-seq) were performed at the Interdisciplinary Center for Biotechnology Research (ICBR), University of Florida (Cytometry, RRID:SCR_019119; Monoclonal Antibody – RRID:SCR_019147; Gene Expression and Genotyping Core, RRID:SCR_019145; NextGen DNA Sequencing Core Facility, RRID:SCR_019152).

Three mice (seven- to eight-weeks-old female BALB/c) were used per antigen group. Before the first immunization, an initial blood sample was collected from a facial vein of each mouse. A subcutaneous injection of 50 μg of nematode extract in Imject Freund's Incomplete Adjuvant (FIA; Thermo Scientific, Rockford IL, USA) was used for immunization, which was repeated three, six, 13 and 17 weeks later. One week after the second and those subsequent immunizations, blood was collected, and serum anti-nematode antibody titers were evaluated using indirect ELISA. Four weeks after the fifth immunization, mice were injected with 25 μg of nematode extract intraperitoneally. Four days later, the mice were sacrificed to harvest their spleens for B-cell enrichment and sorting. Animal use protocols were approved by the University of Florida Institutional Animal Care and Use Committee (IACUC #10682 and #09723).

### B-cell enrichment and flow cytometry

Spleens were processed into single-cell suspensions by grinding the tissues between frosted ends of two glass slides and passing the suspension through a 70-μm filter (Genesee Scientific, San Diego CA, USA) to remove debris. Red blood cells were lysed by incubating the suspension in Ammonium-Chloride-Potassium buffer (ACK; NH_4_Cl 8,024 mg/L, KHCO_3_ 1,001 mg/L, EDTA·Na_2_·2H_2_O 3.722 mg/L) on ice for 15 min, quenching with PBS, and collecting nucleated cells by centrifugation (300 × *g* for 5 min at 4°C). CD19^+^ cells were isolated using Mouse CD19 magnetic MicroBeads and LS columns (Miltenyi Biotec, Auburn CA, USA) according to the manufacturer's protocol.

Cells were resuspended at 1 × 10^6^ concentration in 1 mL solution containing 100 μL BD Brilliant Staining Buffer Plus (BD Biosciences, Franklin Lakes NJ, USA) and a mixture of dye-labelled rat anti-mouse antibodies (CD20, CD3, CD19, BC45R/B220a, IGD and IGG) for cell sorting using flow cytometry. The suspension was incubated at room temperature in the dark for 30 min, after which 1 mL of BD Brilliant Staining Buffer was added and centrifuged at 100 × *g* for 5 min. Cells were resuspended in 500 μL of the staining buffer and run-on BD FACSAria IIU/III upgraded cell sorter (BD Biosciences, San Jose CA, USA) with FSC/SSC and 15 detectors. The run was performed using a 70-μm integrated nozzle. Each sample had 40 U/mL of Ribolock added to protect intact cells from RNAases.

### Single-cell RNA-sequencing and analyses

For single cell sequencing, 10x single cell V(D)J libraries were prepared following the 10x Chromium (10x Genomics, Pleasanton CA, USA) Next GEM Single Cell V(D)J user guide. Briefly, 5,000–12,000 FACS-sorted B220+ cells were loaded at 700–1,200 cells/μL onto the 10x Chromium instrument. After breakdown of the Gel Beads-in-emulsion (GEMs), the barcoded cDNAs were purified with DynaBeads. This was followed by 13 cycles of PCR amplification (98ºC for 45 sec; [98ºC for 20 sec, 67ºC for 30 sec, 72ºC for 1 min] × 6; 72ºC for 1 min). The mouse V(D) J were enriched by amplification with Mouse B cell Mix 1 for six cycles of PCR (98ºC for 45 sec; [98ºC for 20 sec, 67ºC for 30 sec, 72ºC for 1 min] × 5; 72ºC for 1 min), followed by eight cycles of PCR amplification using Mouse B cell Mix 2. After library enrichment involving fragmentation, adaptor ligation and index PCR, followed by a DynaBead cleanup, scRNA-seq was performed on an Illumina HiSeq 3000 platform by pair-end (PE)-sequencing on a single lane at 100 PE (i.e., 2 × 100). The sequences generated were processed using the Chromium Single Cell Gene Expression software suite (San Francisco CA, USA) for demultiplexing, barcode processing, gene counting and visualization.

### Cell-free protein synthesis of mAbs

Selected mAbs were synthesized in vitro using a NEB PURExpress CFPS kit (New England Biosciences, Ipswich MA, USA). For this purpose, heavy- and light-chain DNA templates were modified as outlined in Ojima-Kato et al (2017), fulfilling the template requirements for the PURExpress system. From 5′- to 3-end, DNA templates contained: six Ns; T7 promoter; five Ns; a ribosomal binding site (AAGGAG); five Ns; start codon (ATG); SKIK codons; the mAb sequence (N-terminal to C-terminal; 5′ to 3′); Leucine zipper A or B (for heavy or light chain, respectively); stop codon (TAA); and spacer (six Ns) followed by T7 terminator sequence. Antibodies were synthesized in 2-μL PCR tubes using a GenAmp thermal cycler (Applied Biosystems, Waltham, MA, USA). The reaction mixture was prepared following the recommended protocol for PURExpress, and included NEB disulfide bond enhancers 1 and 2 (1.2 μL each) and 300 ng each of the heavy- and light-chain DNA templates. Protein synthesis was carried out on the thermocycler at 37°C for three hours. Sequence information for these mAb is provided in supplementary document **S1**.

### ELISA validation of select mAbs

Indirect ELISA was used to evaluate mAbs synthesized in vitro. Antigen suspensions were diluted to ~20 μg/mL in PBS, of which 50 μL was used for ELISA plate coating overnight at 4°C. The plate was washed three times using 200 μL of PBS, and blocked by filling the wells with 200 μL blocking buffer and incubating overnight at 4°C. The plate was then washed, and 100 μL of primary antibody was added to all wells containing antigens. Four wells were used as negative controls for the four antigens. After overnight incubation at 4°C, the plate was washed and 100 μL of secondary antibody (Peroxidase-conjugated AffinPure Goat Anti-Mouse IgG+IgM (H+L); Jackson ImmunoResearch; West Grove PA, USA) was added to all wells at 1.6 μg/μL and incubated at 4°C overnight. For detection, the plate was washed and 100 μL of TMB (3,3′,5,5′-tetramethylbenzidine; ThermoFisher, USA) substrate was added to all wells and incubated for 30 min at room temperature. The reaction was stopped by adding 100 μL of stop solution (2 M H_2_SO_4_).

## Results and Discussion

The immune response of mice inoculated with the four nematode-derived antigen pools varied both between and within antigen groups. This variation also did not correlate with the number of booster injections received. For example, three of the six SCN-inoculated mice (two HG type 0 and one HG type 1.2.3.5.6.7) did not show a clear immune response, as reflected in the small number of B cells collected and mAb sequences obtained (Table 1). The remaining three mice (one HG type 0 and two HG type 1.2.3.5.6.7) displayed a stronger response, with a higher number of B cells and corresponding mAb sequences (Table 1). It is worth mentioning that this result was from a repeat experiment, since the first set of SCN-inoculated mice, which were all immunized intraperitoneally, did not show immune response to the antigens — despite repeated immunization efforts — and were thus discarded. In contrast, the SCN-inoculated mice from the second set were all immunized subcutaneously.

**Table 1:** Number of monoclonal antibody (mAb) nucleotide sequences generated per antigen type (HG type and race designation for soybean cyst nematode [SCN; *Heterodera glycines*] and root-knot nematode [RKN; *Meloidogyne incognita*]).

**Antigen type**	**No. V(D) J Spanning Cells**	**Total mAb Sequences**
SCN HG type 0	32	3,978
SCN HG type 0	5	
SCN HG type 0	3,941	
SCN HG type 1.2.3.5.6.7	2	6,183
SCN HG type 1.2.3.5.6.7	2,784	
SCN HG type 1.2.3.5.6.7	3,397	
RKN race 3	8,995	27,098
RKN race 3	10,178	
RKN race 3	7,925	
RKN race 4	8,171	21,313
RKN race 4	13,142	

Mice with antigens from the RKN races 3 and 4 were immunized after completion of sequencing for SCN-inoculated mice; all immunizations with these nematode antigens were administered subcutaneously. Here, again, great variations in immune response were observed within and between mice and antigen groups. ELISA results before spleen harvest showed a clear immune response in both groups of RKN-inoculated mice. However, cell sorting and sequencing library preparation for RKN race 3-inoculated mice showed that there were many individual B cells that produced multiple different antibodies, which would have confounded downstream bioinformatic analyses (data not shown). As a result, these cells were discarded, and a new set of mice was later immunized with this antigen. Individual B cells producing multiple antibody types at the transcriptome level were previously reported in a study by Shi et al (2019), where scRNA-seq was coupled with Sanger sequencing to evaluate repertoires of single B cells, including naïve B cells, memory B cells, and plasma cells. Of the three RKN race 4-inoculated mice, Mouse 3 died before the experiment was completed, while Mouse 2, which had shown the best immune response, was reinoculated two more times for improved response, resulting in ~30,000 B cells that produced single antibodies. A third of these cells were sequenced, while the remaining ~20,000 cells were frozen for later use.

We synthesized two mAbs using cell-free protein synthesis (CFPS) and evaluated their specificity by indirect ELISA. These mAbs were selected from among ~10,000 sequences that were generated using SCN antigens (Table 1). B-cell clonotypes for which there was more than one heavy- and/or one light-chain sequence were excluded; clonotypes with heavy chains lacking sequence information from the diversity (D) region were ignored; and heavy and light chains were considered only when sequence information (≥ 200 nucleotides) was available for the constant regions of these mAb sequences. We also excluded clonotypes with κ-type light chains to reduce the cost of template DNA synthesis, a process involving extension of the constant regions of each antibody fragment with Gibson assembly and cloning of assembled fragments into *E. coli* cells to produce sufficient templates for CFPS, all using NEB's (Ipswich MA, USA) kits and protocols. A total of 84 mAbs of classes IgD (8), IgG (4) and IgM (72) were considered, and two mAb sequences of the IgM class (representing the majority of the 10,000 sequences generated) were randomly picked for CFPS. DNA fragments of the mAb fragments were synthesized at Eurofins (San Ramon CA, USA).

**Figure 1:**
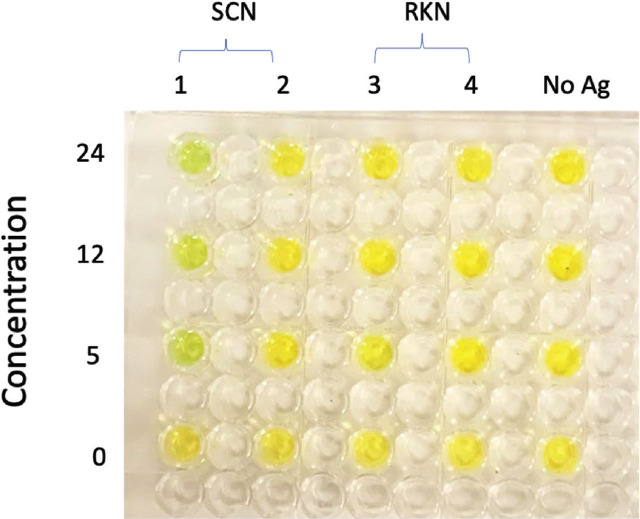
Indirect ELISA showing the interaction of antigen HG type 0-based monoclonal antibody (mAb) against the four nematode antigen types generated from soybean cyst nematode (SCN) and root-knot nematode (RKN): SCN 1 (HG type 0), SCN 2 (HG type 4.5.7), RKN race 3 and RKN race 4. The concentration of the mAb is in proportion to the template DNA used during the protein synthesis and is therefore in ng/μL.

Synthesized mAbs based on sequences from the two SCN antigens (HG type 0 and HG type 1.2.3.5.6.7) were tested against four nematode antigen pools (HG type 0, HG type 4.5.7, RKN race 3 and RKN race 4), as well as a negative control that contained no nematode antigens (Fig. 1). The antigens derived from HG type 1.2.3.5.6.7 were replaced by antigens derived from HG type 4.5.7 in the ELISA test because the former nematode population was no longer available. Again, we note that these phenotype descriptors (HG type and Race) are of populations, with inherent and potentially high variability, such that any specificity or cross-reactivity does not equate to the virulence of the population. However, any mAb specificity has the potential to inform phenotypic species designations. Here, we demonstrate that the mAb that was based on antigen HG type 0 did not react with any of the other three antigens, showing that this mAb was specific to the antigen from derivative population of HG type 0. The second mAb, which was based on HG type 1.2.3.5.6.7, did not react with any of the four antigens, including HG type 0 and HG type 4.5.7 (data not shown). This is an interesting result that highlights the potential use and specificity of these antibodies.

The mAbs we synthesized were in minute quantities, though CFPS is a scalable system (NEB, Ipswich MA, USA). Consequently, we could not test these mAbs on antigens derived from other nematode species, or additional pathotypes. However, our findings serve as a proof of concept that one can generate a vast amount of mAb sequence information using scRNA-seq of mature B cells obtained from nematode antigen-immunized mice, as well as mAb synthesis using CFPS. Moreover, exploiting the use of mAbs in conjunction with ELISA may provide a new avenue for the development of new nematode diagnostic assays that can discriminate between multiple PPN in a soil sample at the species level, but also identify pathotype variations within species.

In general, serological tests like ELISA that use antibody-antigen interactions (protein-based analyses) are simple, rapid and low-cost compared to lab-based PCR tests (DNA-based analyses) and, therefore, have great potential for wide use in agriculture (Bogale et al., 2020; Gong et al., 2021). Of course, there is also a necessity for further research by repeating the experiment with a range of SCN populations, each exhibiting different female index values. For example, if a monoclonal antibody is developed for an HG type 2 population with a female index (FI) of 25 on PI88788, it would also be important to determine whether it maintains its efficacy against an HG type 2 population with a FI of 80 on the same PI88788 background.
